# Key dependent information confidentiality scheme based on deoxyribonucleic acid (DNA) and circular shifting

**DOI:** 10.1016/j.heliyon.2023.e23572

**Published:** 2023-12-12

**Authors:** Sultan Almakdi, Iqra Ishaque, Majid Khan, Mohammed S. Alshehri, Noor Munir

**Affiliations:** a Department of Computer Science, College of Computer Science and Information System, Najran University, Najran, Saudi Arabia; b Department of Applied Mathematics and Statistics, Institute of Space Technology, Islamabad Pakistan

**Keywords:** Chaos, DNA, Image encryption, Logistic map

## Abstract

In this era of advanced information technology, the exploration and development of novel mechanisms to ensure information confidentiality have consistently captivated the attention of upcoming researchers. In this article, we present a pioneering approach that combines DNA sequencing with a four-dimensional (4D) hyperchaotic map to bolster the security of digital information. Our primary focus is on the design of a robust and secure scheme for encrypting color images, leveraging DNA cryptography and hyperchaos. By extracting three distinct DNA sequences, we generate encryption keys through the integration of DNA computing and 4D hyperchaotic maps. Notably, these keys are intricately linked to the plaintext and vary with any alterations in the input. Consequently, the proposed encryption method stands resilient against an array of potential cryptographic attacks. To gauge the algorithm's security, we subject it to rigorous standard statistical analysis. Our findings underscore the efficiency and robustness of the proposed framework, establishing its potential for facilitating secure communication.

## 1 Introduction

DNA cryptography is emerging as an intriguing domain within cryptography. The extraordinary parallelism and exceptional data density inherent to DNA molecules offer unique attributes that can be harnessed for cryptographic purposes, encompassing data encryption, authentication, and digital signatures. Deoxyribonucleic Acid (DNA) serves as the genetic material present across all living organisms, spanning from viruses to intricate humans. It stands as a universal information repository for life forms. DNA is an extensive polymer composed of minute nucleotide units, each consisting of three distinct components. The immense parallelism and extraordinary data density are unique attributes of DNA molecules that can be harnessed for cryptographic purposes such as data encryption, validation, and digital signatures. Deoxyribonucleic Acid (DNA) serves as the genetic material present in all living species, ranging from viruses to intricate humans. It constitutes an information repository fundamental to all life forms. DNA is an elongated polymer composed of minute nucleotide units, each comprising three distinct components.

i. A Nitrogenous Base I
ii. A sugar with five carbon atoms
iii. A Phosphate Group

Based on the type of nitrogenous base, there exist four distinct nucleotides: Adenine (A), Cytosine (C), Thymine (T), and Guanine (G). DNA adopts a double helix structure, comprising two antiparallel strands. Remarkably, utilizing just these four letters, DNA has the capacity to encode the extensive and intricate information of an organism.

In DNA cryptography, computational cycles should be possible by some compound response. These cycles are controlled precisely and produce a succession of nucleotides (for example, A, T, C, and G) as a yield for data encryption. It is worked out by explicit hybridization between the DNA particles. It is shaped by an explicit twofold helix design of a correlative base pair for encoding information and placing it into tasks. Considering ‘TAGC to be a code, we can have 24 combinations that may have the form ‘GCAT,’ ‘CATG,” etc. (4! = 24). Among these 24 combinations, one can have eight possible in real this is because ‘C' can make a pair with ‘G' and ‘A' can make a pair ‘T' due to the properties of DNA [[4], [5], [6]]. Digital DNA coding is executed on a small-scale utilizing DNA nucleotide code encompassing A, C, T, and G. The primary pair operates as a key to modify the nucleotide sequence. This resulting sequence, in the form of an amino acid sequence, is encoded using DNA codes to yield the ultimate ciphertext. This sequence serves to obfuscate the utilization of DNA coding from potential attackers. Consequently, this design presents a user-friendly and secure system grounded in matrix calculation techniques that merge performance with mathematical and biological principles. The foundation of DNA encryption lies within computer-aided DNA concepts. While true DNA encoding is yet to be fully realized due to the possibility of performing chemical reactions in a laboratory setting, the prospect exists. Silicon chips utilized in computers could potentially be supplanted with DNA chips [[7], [8], [9]]. Presently, a DNA encryption system based on a mathematical formulation that expresses data within natural DNA sequences is feasible. This approach renders the data challenging to read and predict.

Chaotic encryption involves encrypting information using the intricate dynamics of chaos. The fundamental concept underlying chaotic encryption revolves around harnessing the intricate behavior of chaotic systems to formulate encryption techniques that are challenging to decipher. The chaotic dynamics that form the basis of the encryption process serve as a wellspring of randomness, imbuing the encryption process with the appearance of randomness and thus enhancing its security. The four-dimensional (4D) Logistic Map is characterized by complex and unpredictable dynamical behavior, boasting a pair of effective Lyapunov exponents within nonlinear systems. This intricate behavior, coupled with uncertainty and randomness, provides logistic systems with a substantially stronger foundation, offering improved safety and performance compared to 1D chaotic systems. In the realm of digital image security, techniques have been established utilizing the chaotic 3D self-exciting single-disk homopolar dynamo [2]. Another encryption scheme built upon the chaotic Rabinovich-Fabrikant system and S8 confusion component has been introduced [3]. Numerous other image encryption schemes grounded in chaos have been documented in the literature [[10], [11], [12], [13], [14]]. A 2D logistic map and Lorenz-Rossler chaotic system-based RGB image encryption approach has been presented by Kumar et al. [18]. A DNA-coded fuzzy-based (DNAFZ) *S*-boxes for image encryption using hyperchaotic map has been presented in Ref. [17]. A similar image encryption approach has been utilized in Refs. [15,16]. Yan et al. presented a chaotic image encryption algorithm based on an arithmetic sequence scrambling model and DNA encoding operation [20]. An encryption method for medical images using 2D Zaslavski map and DNA cryptography Chirakkarottu [21]. DNA cryptography-based Image Encryption Scheme using Multiple Chaotic Maps and SHA-256 hash function has been presented in Ref. [23]. Zhu et al. presented an Image encryption scheme based on chaotic maps and parallel DNA coding [24]. A similar approach has been utilized by Abdelfatah et al. [25]. Xian et al. utilized a double parameters fractal sorting matrix for image encryption [26]. A cryptosystem based on the combination of double parameters fractal sorting vector and spatiotemporal chaotic system has been presented in Ref. [27]. A Boolean network encryption algorithm based on the 2-D chaotic map has been presented in Ref. [27].

Chaotic map-based encryption algorithms are highly resistant to cryptographic attacks, but they are slower due to their intricate structure. Moreover, these cryptosystems are vulnerable to chosen plain text attacks [28,29]. Whereas DNA-based cryptosystems are renowned for having error-correcting features. The total error tolerance of the system can be further improved by combining DNA cryptography with a hyperchaotic map, ensuring that minor mistakes in the DNA sequences or during the encryption process don't result in severe decryption problems. DNA-based processing is characterized by high parallelism, massive data storage, and extensive security.

In response to the above analysis, we have presented a novel cryptosystem based on DNA encoding and 4D hyperchaotic map. In the realm of DNA encoding, both pairs of nucleotides serve as data carriers. Within a cryptosystem, data sequences are generated to facilitate data encryption. The primary step in this encryption process involves converting data—be it text, images, or audio—into binary or another numerical representation. The encryption procedure leverages a range of biological mechanisms, such as DNA fragmentation, DNA chip technology, DNA bio-molecular techniques, One Time Pad (OTP), and Polymerase Chain Reaction (PCR). Additionally, practical functions like XOR and XNOR can also be employed to achieve the desired outcomes. Following this, the conversion of DNA occurs, wherein encrypted data is transformed into a DNA format. We present a novel iteration of the DNA cryptosystem that incorporates DNA chemicals. This involves the implementation of confusion and diffusion functions. The DNA sequence yields the key image. The process further employs a 4D hyperchaotic map for generating a random sequence, subsequently used to scramble the DNA-encrypted data. The scrambled pixels are then transformed into binary form, followed by the application of circular shifting using a designated algorithm. Comprehensive standard security analyses are conducted on the encrypted images produced by our proposed algorithm. The performance analysis indicates that the encrypted images exhibit favorable random properties, with evenly distributed pixel values.

The rest of the work is ordered as follows: Section 2 presents the literature review; Section 3 describes the proposed cryptosystem system described; Performance and security analysis has been presented in Sections 4 and 5. Lastly, the conclusion and future recommendations have been presented.

## 2 Literature review

DNA encryption has found extensive application, particularly in the realm of medical images. The field has witnessed a multitude of robust research endeavors focusing on image-encoding algorithms grounded in DNA principles. In recent times, DNA encryption has been seamlessly integrated with various chaotic systems, resulting in innovative approaches. Diverse chaotic maps have been harnessed to encrypt both grayscale and RGB images, signifying the versatility of this method. Moreover, the marriage of chaotic maps and DNA cryptography has given rise to a plethora of research endeavors aimed at video encryption. The collective body of work showcases the evolving landscape of DNA-based encryption methods and their widespread utilization across various types of multimedia data.

**Table:** 

Reference	Year	Image encryption scheme
Alghafis et al. [1]	2020	Chaos and DNA sequencing
Mou et al. [10]	2019	Hyper-chaotic Map
Javan et al. [11]	2021	Multi-mode Chen Hyper chaotic map
Karamkar et al. [12]	2021	Video encryption using Hyperchaos and DNA
Zhou et al. [13]	2021	Conservative hyperchaotic systems and closed-loop diffusion
Elkamchouchi et al. [14]	2020	DNA confusion and Hybrid chaotic map diffusion
Özkaynak et al. [15]	2014	Hyper-chaotic system and DNA sequence operation
Mohamed et al. [16]	2021	DNA coded using Hyper chaotic map
Kumar et al. [17]	2021	2D Logistic map
Surenda et al. [18]	2014	Random Key Generation scheme
Iqbal et al. [19]	2011	Random key generation and hyperchaos

## 3 Design of the proposed scheme

This section outlines the architecture of our proposed encryption design. To facilitate an understanding of the encryption and decryption processes inherent to our scheme, we have introduced fundamental terminologies in the subsequent section.

### 3.1 Some basic terminologies

#### 3.1.1 4-D hyper chaotic map

Chaos is a ubiquitous phenomenon in deterministic nonlinear systems that show sensitivity to the original state and have random behavior. Since chaotic maps are consistent, time-varying flexibility systems, they can produce a series of pseudorandom series. Sensitivity to initial conditions, ergodicity, and randomness are the properties of chaos. Due to this reason, it has generally been used in image encryption. The 4-D hyperchaotic map is given by.

(1) $$\begin{array}{l} {\dot{x}}_{1}=a\left(x_{2}-x_{1}\right)\text{,}\\ {\dot{x}}_{2}=x_{1}x_{3}+x_{4}\text{,}\\ {\dot{x}}_{3}=b-x_{1}x_{2}\text{,}\\ {\dot{x}}_{4}=x_{2}x_{3}-c_{2}x_{4} \end{array}$$

where a,b,c are real constant parameters, $x_{1},x_{2},x_{3},x_{4}$ are state variables, and a=40,b=2,c=22 [30]. Compared to low-dimensional maps, high-dimensional maps (especially chaotic systems) have greater keyspace, better sensitivity, complex flexibility, and random features. Low-dimensional maps can be categorized using phase space reconstruction and nonlinear prediction techniques, but those processes cannot specify high-dimensional programs.

### 3.2 Encryption scheme

The proposed encryption scheme is organized into four distinct phases. In the initial phase, the positions of the image's pixels are shuffled utilizing a chaotic sequence. Moving forward, the shuffled image matrix is transformed into a DNA sequence during the subsequent phase. Following this, the DNA sequence matrix is perturbed through the amalgamation of a chaotic sequence alongside DNA XOR and DNA multiplication. Finally, the ultimate encrypted image is derived through DNA decoding and subsequent recombination. This multi-step process ensures the security and integrity of the data while preserving the core attributes of the original image.

Step 1 **Pixels conversion** into **binary**

An original image P whose size is m×n×3 is taken, whose pixel range is from 0 to 255. The pixels of the image are converted to binary form, and a matrix is obtained.

Step 2 Separation into R, G, B

Image pixels of red, green, and blue colors are separated.

Step 3 Obtaining Key

Key K is obtained from the DNA sequence, then for each $K_{i}\in K,{and\;P}_{j}\in P\text{,}$.

$$\left\{ \begin{array}{l} K_{i}\times P_{j},If\gcd\left(P_{j},256\right)=1,\\ K_{i}⊕P_{j}If\gcd\left(P_{j},256\right)\neq 1. \end{array}\right.$$

The basic structure of DNA consists of four nucleotide elements, namely, Adenine (A), Cytosine (C), Guanine (G), and Thymine (T). These nucleic acid bases are encoded as G = 10, C = 01, A = 00, and T = 11, respectively. The DNA XOR operation and multiplication are presented in Table 1, Table 2.

**Table 1:** DNA XOR operation.

⊕	A	T	C	G
A	A	T	C	G
T	T	A	G	C
C	C	G	A	T
G	G	C	T	A

Here A = 00, and C = 01, so 00⊕01=01, where C = 01.

**Table 2:** DNA Multiplication operation.

×	A	T	C	G
A	T	A	G	C
T	A	T	C	G
C	G	C	T	A
G	C	G	A	T

**Fig. 1:**
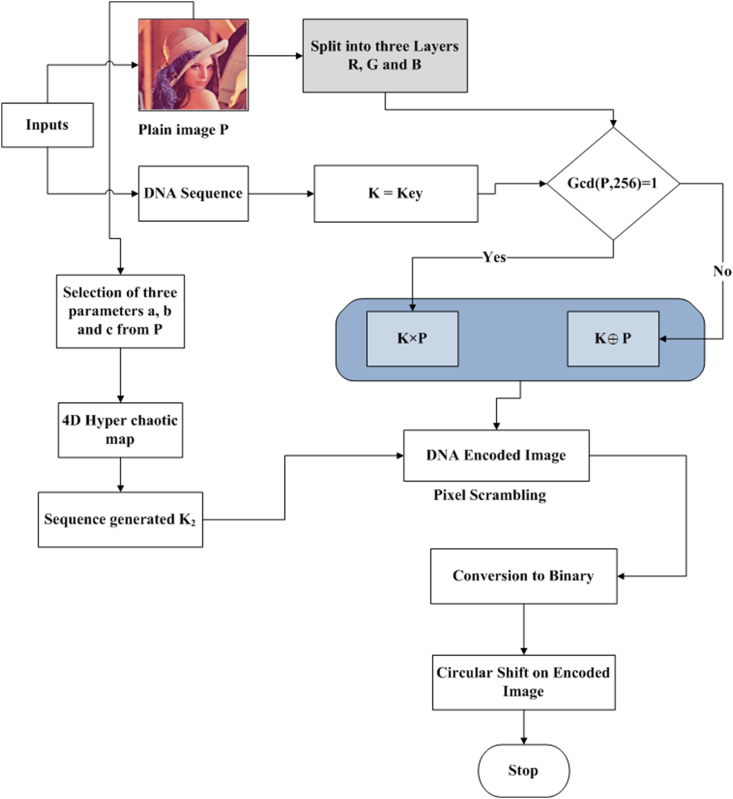
Flow chart of offered design.

The design of our suggested encryption scheme is presented in Fig. 1.

Step 4 Iterating a 4D hyperchaotic map

The 4D hyperchaotic map is repeated N times to enhance the security and remove the adverse effect. Then the system is repeated m×n times with control parameters =40,b=2,c=22 , and initial values are considered to produce chaotic sequences. The 4D hyperchaotic map has been utilized to produce a random sequence.

Step 5 Scrambling

DNA-encoded images obtained from Step 3 are scrambled using a random sequence generated by a 4D hyperchaotic map.

Step 6 Circular shifting

These scrambled pixels are converted to binary, and then circular shifting is applied using the algorithm given in the next section.

Step 7 Encrypted image

The matrices obtained in the previous step are compiled as encrypted images.

DNA XOR works in the following manner.

**Fig. 2:**
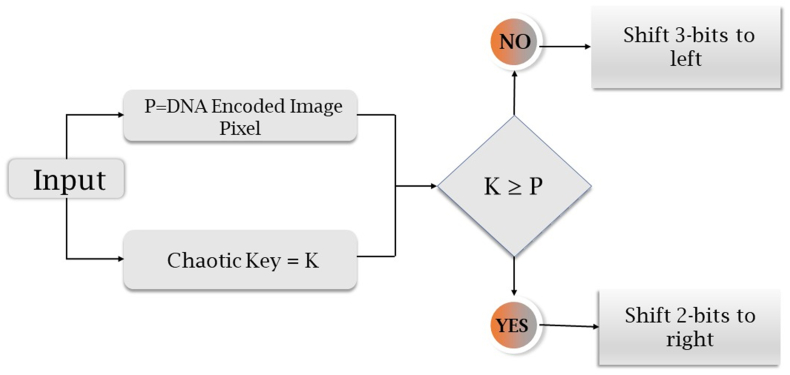
Pseudo-code for circular shifting.

**Fig. 3:**
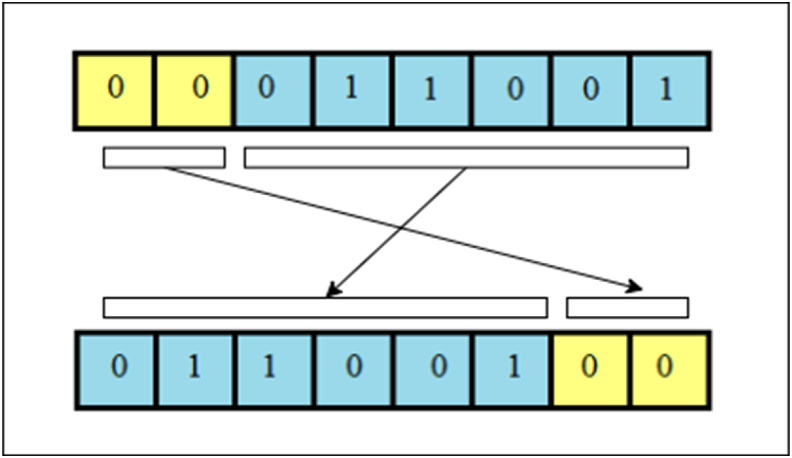
Right circular shift of two bits.

Algorithm for Circular shifting:

We will apply circular shifting on DNA encoded image by comparing the value of each pixel with the chaotic key value by using the following rule:Fig. 2, Fig. 3.

Example:

To understand the algorithm for circular shifting, we have presented an example.

Let chaotic key be $\left[\begin{array}{cc} 45 & 24\\ 9 & 22 \end{array}\right]$ and DNA encoded image be $\left[\begin{array}{cc} 25 & 6\\ 17 & 21 \end{array}\right]$. Then DNA encoded matrix in binary form is $\left[\begin{array}{cc} 00011001 & 00010110\\ 00010001 & 00010101 \end{array}\right]$.

**Rule 1:** As chaotic key-value ≥ DNA encoded image pixel, i.e., 45>25, we will shift 2 bits to the right in the DNA encoded matrix.

In the binary form of 25=00011001, two bits 00 have been shifted circularly towards the right, giving a new pixel value 01,100,100, representing 100 in decimal form.

**Rule 2:** As chaotic key-value < DNA encoded image pixel, i.e. 9<17, we will shift 3 bits to the left in the DNA encoded matrix.

In the binary form of 17=00010001, three bits 001 have been shifted circularly towards the left, giving a new pixel value 00100,010, representing 34 in decimal form.

## 4 Performance analysis

This section comprises performance analyses of our proposed technique. We have performed some standard analysis to check the security and robustness of our scheme.

### 4.1 Histogram analysis

Encrypted image histogram analysis is one of the most straightforward methods for characterizing image encryption. The proposed scheme has been utilized to convert plain images into encrypted images. MATLAB 2019-a is used to execute the simulation. The layer-wise histograms of the plain image and the histograms of the respective enciphered images are represented in Fig. 5. The histogram of the encrypted image is considerably altered from the corresponding plain image and is evenly distributed.

### 4.2 Correlation analysis

This analysis gives the measure of correlation among the neighboring pixels in images that were given digitally. Combination measures the level of contact between two pixels. A low level of interlocking pixels indicates the robustness of the encryption process. Pearson's correlation coefficient is measured as:

(2) $$\gamma_{xy}=\frac{E\left(\left(x-\mu_{x}\right)\left(y-\mu_{y}\right)\right)}{\sqrt{\delta_{x}\delta_{y}}}\text{,}$$

where μ is the expected value, and δ is the variance. The result obtained from correlation analysis is presented in Table 3. The correlation values presented in Table 3 show the vulnerability of our scheme to differential attacks. The scheme meets the standard criteria as the value of correlation is very close to zero.

**Table 3:** Correlation analysis of original and enciphered images.

Images	Layers	Original image			Encrypted image		
		D	H	V	D	H	V
Lena	R	0.9451	0.9526	0.9763	−0.0023	0.0014	0.0052
	G	0.9293	0.9373	0.9698	−0.0043	−0.0075	0.0014
	B	0.9293	0.9236	0.9543	−0.0036	−0.0071	0.0042
Baboon	R	0.9083	0.9448	0.9185	0.0024	0.0020	0.0024
	G	0.9014	0.8706	0.8330	0.0018	0.0048	−0.0033
	B	0.8767	0.9212	0.9116	0.0033	−0.0003	−0.0015
House	R	0.9216	0.9642	0.9326	−0.0018	−0.0040	0.0021
	G	0.9126	0.9760	0.9409	−0.0005	−0.0020	0.0011
	B	0.9317	0.9777	0.9668	−0.0017	0.0014	0.0020
Fruits	R	0.9627	0.9777	0.9505	0.0059	−0.0053	0.0019
	G	0.9402	0.9583	0.9554	0.0017	−0.0051	0.0022
	B	0.9436	0.9578	0.9601	−0.0034	−0.0045	0.0039
Peppers	R	0.9405	0.9630	0.9660	0.0004	−0.0011	−0.0001
	G	0.9500	0.9664	0.9706	0.0017	−0.0025	0.0023
	B	0.9290	0.9524	0.9592	−0.0007	−0.0062	0.0053
Airplane	R	0.9290	0.9389	0.9220	0.0017	−0.0025	0.0049
	G	0.8808	0.9200	0.9336	0.0002	0.0004	0.0041
	B	0.8749	0.9495	0.8977	0.0013	0.0030	0.0058

**Fig. 4:**
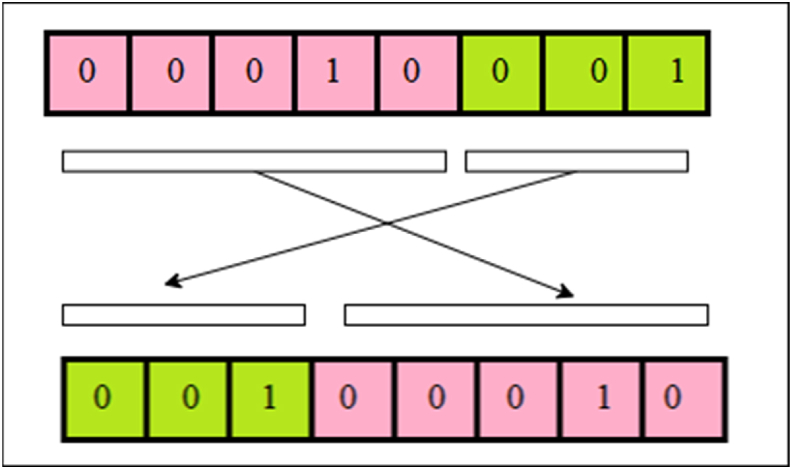
Left circular shift of three bits.

Fig. 4 represents the correlation diagrams in the diagonal, horizontal, and vertical directions of original images and their corresponding enciphered images (see Fig. 3). It is evident from Fig. 6 that correlation is uniformly distributed between two neighboring pixels of the enciphered image. In contrast, neighboring pixels of a plain image are extremely correlated.

**Fig. 5:**
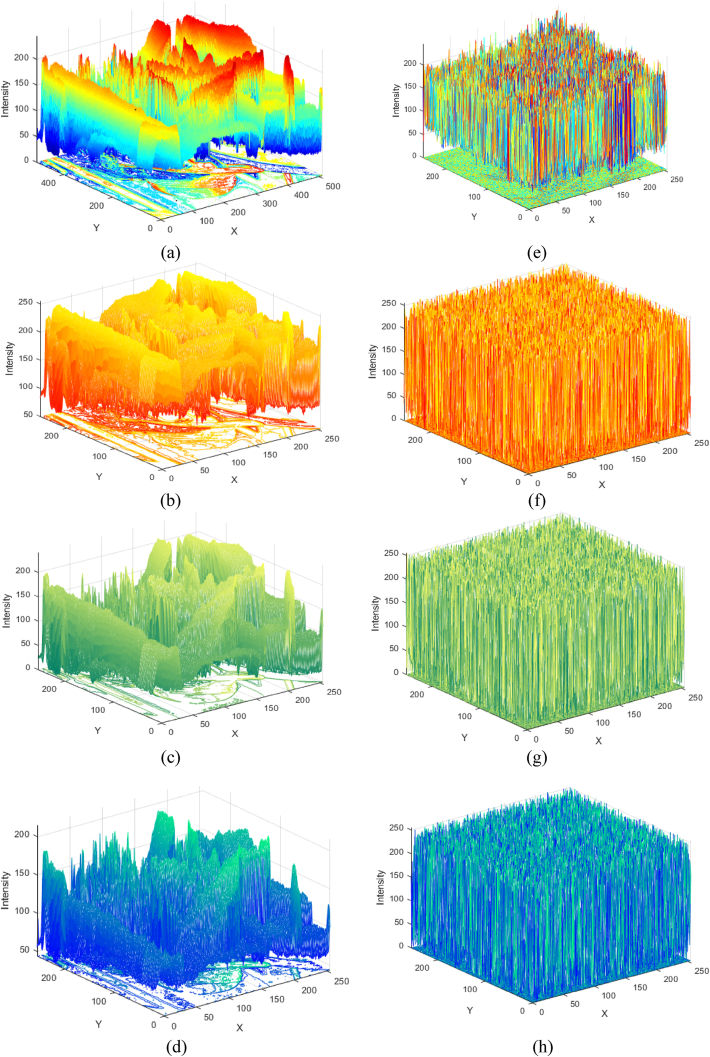
Histograms of Lena: (a) original color image, (b)–(d) original image red, green, and blue layer, (e) encrypted color image, (f)–(h) encrypted red, green, and blue layer.

**Fig. 6:**
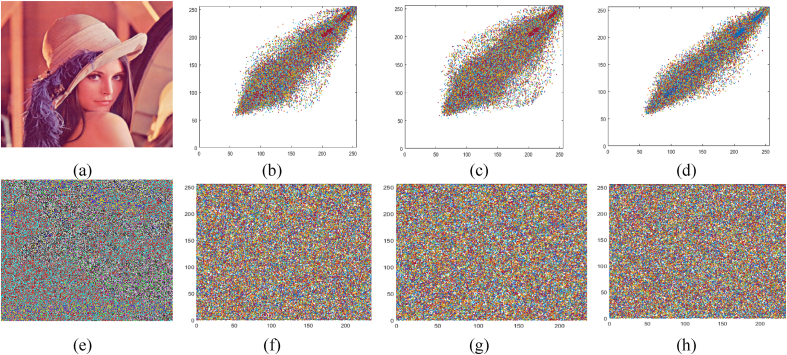
Correlation diagram of Lena image (a) original image (b)–(d) vertical, diagonal and horizontal original image, (e) encrypted image, (f)–(h) vertical, diagonal and horizontal encrypted image.

Table 4 represents the comparative analysis of our proposed scheme and scheme proposed in Ref. [3]. The results of Table 4 show that our proposed scheme provides enhanced image encryption.

**Table 4:** Comparative analysis of Correlation of proposed scheme and Reference.

Images	Layers	Ref [2]			Proposed scheme		
		D	H	V	D	H	V
Lena	Red	0.0032	0.0011	0.0014	−0.0023	0.0014	0.0052
	Green	−0.0028	0.0005	0.0016	−0.0043	−0.0075	0.0014
	Blue	0.0021	0.0021	−0.0001	−0.0036	−0.0071	0.0042
Baboon	Red	0.0005	−0.0038	0.0010	0.0024	0.0020	0.0024
	Green	−0.0002	0.0006	0.0015	0.0018	0.0048	−0.0033
	Blue	0.0005	−0.0008	−0.0019	0.0033	−0.0003	−0.0015
House	Red	−0.0042	−0.0042	0.0041	−0.0018	−0.0040	0.0021
	Green	0.0017	−0.0006	0.0022	−0.0005	−0.0020	0.0011
	Blue	−0.0019	0.0003	−0.0022	−0.0017	0.0014	0.0020
Fruits	Red	−0.0001	0.0013	0.0026	0.0059	−0.0053	0.0019
	Green	−0.0026	0.0034	0.0020	0.0017	−0.0051	0.0022
	Blue	0.0000	−0.0000	−0.0007	−0.0034	−0.0045	0.0039
Peppers	Red	−0.0000	0.0010	0.0007	0.0004	−0.0011	−0.0001
	Green	0.0000	−0.0006	0.0006	0.0017	−0.0025	0.0023
	Blue	−0.0008	−0.0002	0.0014	−0.0007	−0.0062	0.0053
Airplane	Red	0.0030	0.0011	−0.0013	0.0017	−0.0025	0.0049
	Green	−0.0014	0.0030	0.0016	0.0002	0.0004	0.0041
	Blue	−0.0021	−0.0017	−0.0004	0.0013	0.0030	0.0058

### 4.3 Information entropy

It gives the measure of uncertainty within an image and can be used to illustrate the inconsistency of image information. Which can be calculated in the following relationships:

(3) $$I\left(m\right)=\sum_{i=0}^{2^{N}-1}P\left(m_{i}\right)Log_{2}\frac{1}{P\left(m_{i}\right)}\text{,}$$

Here $m_{i}$ is a gray level with $P\left(m_{i}\right)$ is the probability density function. In an enciphered image, an entropy value closer to 8 shows the highest uncertainty in the enciphered image and the performance of the existing scheme. The values of entropy for enciphered images have been presented in Table 5. The result obtained in Table 5 depicts that the entropy value of encrypted images is nearer to the ultimate value, which depicts the security of our proposed design against entropy attempts.

**Table 5:** Entropy analysis of the plain and encrypted image.

Name of image	Original	Encrypted	Ref [2]	Ref [3]
Lena	7.2283	7.9988	7.7134	7.9588
Baboon	7.3626	7.9992	7.7624	–
House	7.4810	7.9991	7.0686	–
Fruits	7.4902	7.9991	7.4515	–
Peppers	7.5589	7.9991	7.6698	7.9590
Airplane	6.7056	7.9991	6.6639	–

### 4.4 Differential attacks

A common feature of image encryption should possess high sensitivity to slight changes in original images. The differential analysis enables the attacker to make small variations to the plain image and update the enciphered image. NPCR (number of the pixel exchange rate) and UACI (Unified average Changing Intensity) measure the effect of changing the pixel in the clear image and the corresponding encrypted image.

NPCR and UACI are the determinants used to calculate the resistance to pixel transition rates in a separate attack. The pixel transition rate can be obtained using NPCR described as:

(4) $$NPCR=\frac{\sum_{m,n}D_{1}\left(m,n\right)}{w_{1}\times H_{1}}\times 100\%\text{,}$$

where $H_{1}$ , W are height and width of the image $D_{1}\left(m,n\right)$ is written as:

(5) $$D_{1}\left(m,n\right)=\left\{ \begin{array}{l} 0,ifI_{ee}\left(m,n\right)=I_{e}\left(m,n\right)\\ 1,fI_{ee}\left(m,n\right)\neq I_{e}\left(m,n\right) \end{array}\right.$$

Let $I_{e}$ and $I_{ee}$ represent the encrypted images obtained from a plain image, then UACI (Unified Average Changing Intensity) is defined as:

(6) $$UACI=\frac{1}{W_{1}+H_{1}}\left[\sum_{m,n}\frac{\left|I_{ee}\left(m,n\right)-I_{e}\left(m,n\right)\right|}{255}\right]\times 100\%$$

The NPCR and UACI values of all images are represented in Table 6. These values are greater than the critical values, which indicates that our image encryption scheme is very sensitive to slight deviations in original images and has great potential for contrary differential cryptanalysis. We have also compared our values with the encryption scheme proposed in Ref. [2], which shows our encryption is better for image encryption.

**Table 6:** Comparative analysis of NPCR and UACI results.

Proposed scheme			Ref. [2]	
Image	UACI	NPCR	UACI	NPCR
Lena	33.41	99.61	33.45	99.59
Baboon	33.52	99.65	33.46	99.61
House	33.40	99.64	33.42	99.62
Fruits	33.44	99.85	33.46	99.60
Peppers	33.61	99.68	33.44	99.58
Airplane	34.01	99.25	33.49	99.63

### 4.5 Security analysis

Robust encryption must pose a large keyspace enough to allow for illegal attacks. A good encryption scheme must be highly sensitive to secret keys. Test results also show that our system is highly sensitive to private key conflicts.

#### 4.5.1 Key sensitivity and keyspace analysis

Every encryption scheme should be sensitive to the secret key. A minor change in the key may fail deciphering and other processes. The level at which the ciphertext changes when the first key changes slightly refer to the critical sensitivity. When a turbulent system changes the original value slightly, there will be a significantly different reconstructed image.

**Fig. 7:**
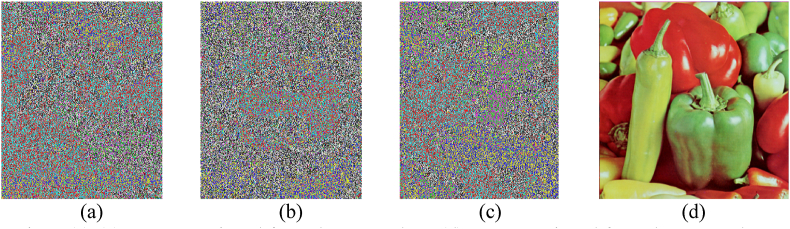
(a)–(c) Images retrieved from the wrong key, (d) Image retrieved from the correct key.

We can observe from Fig. 7 that the original image can't be retrieved from the wrong key, the plain image can only be retrieved from the actual key.

#### 4.5.2 Brute force attack

A widely recognized and potent form of attack, often labeled as a “brute force attack,” exploits the principle of systematically attempting various permutations of a target password until the correct password is identified. The number of trials required depends on the password's length, demanding more attempts for longer passwords. Such non-violent attacks can be time-intensive, yet in instances of feeble passwords, success can be achieved within seconds, without significant exertion. Brute force attacks are straightforward and dependable in their approach. Attackers delegate computational tasks to a computer, like attempting diverse combinations of usernames and passwords until a valid one is discovered. Mitigating and countering these aggressive attacks is pivotal. Rapidly identifying and halting such attempts is crucial. Once attackers gain access to a network, the challenge of apprehending them escalates considerably. Preventing their initial entry is of utmost importance, as once they breach the network, their detection and apprehension become markedly more challenging. If a cryptosystem has a key space of more than $2^{100}$, then it becomes resistant to brute force attack [31]. In our proposed encryption algorithm, the key space is $2^{245}$ which is larger than $2^{100}$. As a result, the suggested scheme's key space is large enough to effectively resist brute force attacks.

#### 4.5.3 Time complexity analysis

The run time of an encryption algorithm is very important. The more amount of time is consumed by an encryption algorithm it is very difficult to implement it in applications. The time of encryption increases with the increase in the size of the image. We have presented the time complexity analysis of our scheme in Table 7 and compared it with existing ones.

**Table 7:** Time complexity analysis of proposed scheme and Reference Unit (s).

	Proposed	[32]	[33]
Lena (128×128×49)	0.4901	0.494	–
Baboon (128×128×64)	0.474	0.478	–
Lena (256×256×256)	0.576	0.606	0.031
Baboon (512×512×3)	0.1131	–	0.1033

### 4.6 Known and chosen plain text attacks

In every encryption system, ensuring robust security capabilities remains a paramount concern. The evolution of attack methodologies aims to unearth vulnerabilities within established encryption systems. This continuous interplay between protective measures and attack techniques drives the refinement of both encryption and intrusion methods, ultimately leading to the development of more resilient systems. Various approaches are employed to challenge image encryption, including known-plaintext attacks (KPA), selected plaintext attacks (CPA), and ciphertext-only attacks (COA). In a CPA scenario, the attacker can interact with the encryption system, influencing the explicit content that is input. Security keys are derived based on the chosen pairs of plaintexts and their corresponding ciphertexts. A chosen plaintext attack represents a cryptanalysis attack model where the assailant possesses the capability to select arbitrary plaintexts, subsequently observing the corresponding ciphertexts. This iterative process of probing and strengthening creates a dynamic landscape wherein encryption systems evolve to counteract emerging threats.

Our suggested cryptosystem takes advantage of the capabilities of DNA encoding and chaotic maps to construct a strong and secure encryption mechanism. The complex dynamics of the 4D hyperchaotic map and the complicated character of DNA-encoded data both contribute to the cryptosystem's robustness against known-plaintext and chosen-plaintext attacks. This novel approach demonstrates the possibility of novel techniques in improving the security landscape of cryptographic systems.

### 4.7 Noise attack

#### 4.7.1 MSE

It is used to show the combined square error between the recovered image and the plain image. The accuracy of any algorithm can be determined based on these metrics including the alteration between the original image and enciphered image. Lesser errors are reflected in the lower value of MSE. The following formula can estimate MSE:

(7) $$MSE=\frac{1}{MN}\sum_{P=1}^{M}\sum_{Q=1}^{N}{\left(F\left(P,Q\right)-I\left(P,Q\right)\right)}^{2}\text{,}$$

where P,Q refers to the dimensions of the image.

#### 4.7.2 PSNR

It is the ratio of high signal strength and destructive noise effects that affect signal precision. Peak signal-to-noise ratio (PSNR) can be estimated by a mean square error (MSE), as shown in the following relation:

(8) $$PSNR=10\log_{10}\left(\frac{L^{2}}{MSE}\right)\text{,}$$

Where L is the value of the range of grayscale in the image. L=256 for an 8 bit image. It is found that the higher the PSNR, the lower the distortion.

Table 8 represents the MSE and PSNR values of layers of original and corresponding plain images. These values suggest that the offered structure has resistance against all the attacks related to MSE and PSNR.

**Table 8:** MSE and PSNR results.

MSE				PSNR		
Image name	R	G	B	R	G	B
Lena	10623.15	9074.28	7071.34	7.90	8.59	9.67
Baboon	8321.94	7311.33	9097.10	8.96	9.52	8.58
House	6801.11	8589.94	9557.95	9.84	8.82	8.36
Fruits	11130.70	9887.52	9075.28	7.70	8.21	8.59
Peppers	7929.56	11188.59	11035.32	9.17	7.68	7.74
Airplane	9901.09	10574.99	10409.21	8.21	7.92	7.99
[22] Lena	10663.29	8982.02	7021.32	7.8518	8.5970	9.66
[22] Peppers	8032.50	11143.31	11101.16	9.08	7.66	7.67

## 5 Conclusion and future recommendations

This study introduces a novel encryption scheme tailored for RGB images, rooted in the realms of DNA cryptography and 4D hyperchaos. The outcomes yielded from entropy information and correlation analyses indicate the scheme's efficacy in efficiently encrypting plain images. Furthermore, the calculated values of NPCR (Normalized Pixel Change Rate) and UACI (Unified Average Changing Intensity) affirm the scheme's robustness against diverse attacks. Through comparison with existing schemes, our encryption approach emerges as both secure and resilient. Notably user-friendly, our proposed scheme demonstrates its adeptness in thwarting various cryptographic attacks. The culmination of our results underscores the robustness of our encryption scheme in ensuring the secure transmission of images. Building on this foundation, the presented scheme can be adapted for the encryption of multimedia data such as videos and audio. By segmenting these media formats into smaller units like frames for videos and audio segments for audio files, individual data chunks can be encrypted, signifying the adaptability and extensibility of our proposed approach.

## Ethical approval

This article does not contain any studies with human participants or animals performed by any of the authors.

## Funding statement

The authors are thankful to the Deanship of Scientific Research at 10.13039/501100005911Najran University for funding this work under the Research Group Funding program grant code (NU/RG/SERC/12/3).

## Data availability statement

The authors declared that the data will be available on request.

## CRediT authorship contribution statement

**Sultan Almakdi:** Methodology, Funding acquisition, Data curation, Conceptualization. **Iqra Ishaque:** Writing - original draft, Software, Resources, Methodology, Investigation. **Majid Khan:** Writing - review & editing, Validation, Supervision, Resources, Project administration. **Mohammed S. Alshehri:** Validation, Resources, Methodology, Investigation. **Noor Munir:** Visualization, Validation, Methodology, Data curation, Conceptualization.

## Declaration of competing interest

The authors declare that they have no known competing financial interests or personal relationships that could have appeared to influence the work reported in this paper.

## References

[1] Standard, Data Encryption, Data encryption standard, Federal Information Processing Standards Publication 112 (1999).

[2] Munir N., Khan M., Wei, Wej Z., Hussain I. (2020).

[3] Alghafis A., MunirM N., Khan (2020).

[4] Joan D., Rijmen V. (2001). Reijndael: the advanced encryption standard. Software Tools for the Profession. Program.r.

[5] Akkasaligar P.T., Biradar S. (2020). Selective medical image encryption using DNA cryptography. Inform. Security J..

[6] Chirakkarottu S., Mathew S. (2020). A novel encryption method for medical images using 2D Zaslavski map and DNA cryptography. SN Appl. Sci..

[7] Alghafis A., Firdousi F., Khan M., Batool S.I., Amin M. (2020). An efficient image encryption scheme based on chaotic and deoxyribonucleic acid sequencing. Math. Comput. Simulat..

[8] Munir N., Khan M., Wei Z., Akgul A., Amin M., Hussain I. (2020).

[9] Khan M., Munir N. (2019). A novel image encryption technique based on generalized advanced encryption standard based on field of any characteristic. Wireless Pers. Commun..

[10] Alghafis A., Munir N., Khan M. (2021). An encryption scheme based on chaotic Rabinovich-Fabrikant system and S 8 confusion component. Multimed. Tool. Appl..

[11] Jun M., Yang F., Chu R., Cao Y. (2019).

[12] Javan A.A.K., Jafari M., Zare A., Khodatars M., Ghassemi N., Alizadehsani R., Gorriz J.M. (2021). Medical images encryption based on adaptive-robust multi-mode synchronization of chen hyper-chaotic systems. Sensors.

[13] Karmakar J., Pathak A., Debashi N., Mandal M.K. (2021). Sparse representation based compressive video encryption using hyper-chaos and DNA coding. Digit. Signal Process..

[14] Zhou M., Wang C. (2020). A novel image encryption scheme based on conservative hyperchaotic system and closed-loop diffusion between blocks. Signal Process..

[15] ElKamchouchi D.H., Mohamed H.G., Moussa K.H. (2020). A bijective image encryption system based on hybrid chaotic map diffusion and DNA confusion. Entropy.

[16] Özkaynak F., Yavuz S. (2014). Analysis and improvement of a novel image fusion encryption algorithm based on DNA sequence operation and hyper-chaotic system. Nonlinear Dynam..

[17] Mohamed A.G., Korany N.O., El-Khamy S.E. (2021). New DNA coded fuzzy based (DNAFZ) S-boxes: application to robust image encryption using hyper chaotic maps. IEEE Access.

[18] Kumar V., Girdhar A. (2021). A 2D logistic map and Lorenz-Rossler chaotic system based RGB image encryption approach. Multimed. Tool. Appl..

[19] Iqba N., Hanif M., Abbas S., Khan M.A., Rehman Z. (2021). Dynamic 3D scrambled image based RGB image encryption scheme using hyperchaotic system and DNA encoding. J. Inf. Secur. Appl..

[20] Yan X., Wang X., Xian Y. (2021). Chaotic image encryption algorithm based on arithmetic sequence scrambling model and DNA encoding operation. Multimed. Tool. Appl..

[21] Chirakkarottu S., Mathew S. (2020). A novel encryption method for medical images using 2D Zaslavski map and DNA cryptography. SN Appl. Sci..

[22] Alexan W., ElBeltagy M., Aboshousha A. (2022). Rgb image encryption through cellular automata, s-box and the lorenz system. Symmetry.

[23] Rahul B.K., Kuppusamy K., Senthilrajan A. (2023). Dynamic DNA cryptography-based image encryption scheme using multiple chaotic maps and SHA-256 hash function. Optik.

[24] Zhu S., Deng X., Zhang W., Zhu C. (2023). Image encryption scheme based on newly designed chaotic map and parallel DNA coding. Mathematics.

[25] Abdelfatah R.I., Saqr H.M., Nasr M.E. (2023). An efficient medical image encryption scheme for (WBAN) based on adaptive DNA and modern multi chaotic map. Multimed. Tool. Appl..

[26] Xian Y., Wang X., Teng L. (2021). Double parameters fractal sorting matrix and its application in image encryption. IEEE Trans. Circ. Syst. Video Technol..

[27] Xian Y., Wang X., Teng L., Yan X., Li Q., Wang X. (2022). Cryptographic system based on double parameters fractal sorting vector and new spatiotemporal chaotic system. Inf. Sci..

[28] Qi L., Wang X., Ma B., Wang X., Wang C., Gao S., Shi Y. (2021). Concealed attack for robust watermarking based on generative model and perceptual loss. IEEE Trans. Circ. Syst. Video Technol..

[29] Alshehri M., Almakdi S., Qathrady M.A., Ahmad J. (2023). Cryptanalysis of 2D-SCMCI hyperchaotic map based image encryption algorithm. Comput. Syst. Sci. Eng..

[30] Liu Y., Li M., Fan H. (2023).

[31] Alvarez G., Li S. (2006). Some basic cryptographic requirements for chaos-based cryptosystems. Int. J. Bifurc. Chaos.

[32] Gao X., Mou J., Banerjee S., Cao Y., Xiong Li, Chen X. (2022). An effective multiple-image encryption algorithm based on 3D cube and hyperchaotic map. J. King Saud Univ.-Computer and Inform. Sci..

[33] Xu Q., Sun K., Cao C., Zhu C. (2019). A fast image encryption algorithm based on compressive sensing and hyperchaotic map. Opt Laser. Eng..

